# Skeletal effects of RME in the transverse and vertical dimensions of the nasal cavity in mouth-breathing growing children

**DOI:** 10.1590/2177-6709.22.4.061-069.oar

**Published:** 2017

**Authors:** Mario Cappellette, Lucia Hatsue Yamamoto Nagai, Raquel Mori Gonçalves, Aparecida Keiko Yuki, Shirley Shizue Nagata Pignatari, Reginaldo Raimundo Fujita

**Affiliations:** 1 Universidade Federal de São Paulo, Departamento de Otorrinolaringologia e Cirurgia de Cabeça e Pescoço (São Paulo/SP, Brasil).; 3 Private practice (São Paulo/SP, Brasil).

**Keywords:** Palatal expansion technique, Maxilla, Mouth-breathing.

## Abstract

**Introduction:**

Maxillary constriction is a dentoskeletal deformity characterized by discrepancy in maxilla/mandible relationship in the transverse plane, which may be associated with respiratory dysfunction.

**Objective:**

The objective of this study was to evaluate the skeletal effects of RME on maxillary and nasal transverse dimensions and compare the differences between males and females.

**Methods:**

Sixty-one mouth-breathers patients with skeletal maxillary constriction (35 males and 26 females, mean age 9.6 years) were included in the study. Posteroanterior (PA) radiographs were taken before expansion (T_1_) and 3 months after expansion (T_2_). Data obtained from the evaluation of T_1_ and T_2_ cephalograms were tested for normality with the Kolmogorov-Smirnov method. The Student’s *t*-test was performed for each measurement to determine sex differences.

**Results:**

RME produced a significant increase in all linear measurements of maxillary and nasal transverse dimensions.

**Conclusions:**

No significant differences were associated regarding sex. The RME produced significant width increases in the maxilla and nasal cavity, which are important for treatment stability, improving respiratory function and craniofacial development.

## RESUMO

**Introdução:**

a constrição maxilar é uma alteração dentoesquelética relacionada à diminuição transversal da arcada superior, que pode correlacionar-se com problemas respiratórios. O tratamento nos pacientes em crescimento inclui a expansão rápida da maxila (ERM). A correção precoce resulta em maiores alterações esqueléticas e estabilidade dos resultados, podendo evitar desvios de crescimento facial.

**Objetivo:**

avaliar comparativamente, por meio de telerradiografias posteroanteriores (PA), as alterações dimensionais da cavidade nasal pré- e pós-ERM em pacientes respiradores bucais dos sexos masculino e feminino.

**Métodos:**

realizou-se o estudo de medidas lineares em telerradiografias PA pré- e pós-ERM de uma amostra composta por 61 pacientes (35 do sexo masculino e 26 do feminino), com média de idade de 9,6 anos. Todos os pacientes eram respiradores bucais com constrição maxilar esquelética, e foram tomadas radiografias PA pré- e pós-ERM (3 meses). Os dados obtidos foram avaliados nos tempos T_1_ e T_2_ a normalidade dos dados foi confirmada por meio do teste de Kolmogorov-Smirnov. O teste *t* de Student foi aplicado para cada mensuração, para determinar diferenças entre os sexos.

**Resultados:**

a ERM promoveu aumento significativo em todas as medidas lineares maxilares e das dimensões da cavidade nasal.

**Conclusão:**

nenhuma diferença significativa foi associada ao sexo. A ERM produziu aumentos significativos na largura maxilar e volume da cavidade nasal, que são importantes na estabilidade do tratamento Nos pacientes em crescimento e respiradores bucais com constrição maxilar esquelética da maxila, a ERM promoveu aumento do volume da cavidade nasal que melhorou o fluxo aéreo e possibilitou reconduzir o crescimento facial. As alterações transversais não foram significativas quando relacionadas ao sexo.

## INTRODUCTION

Maxillary constriction is a narrowing of the upper arch and is one of the most prevalent malocclusions. Some of its typical features are unilateral or bilateral posterior crossbite, anterior dental crowding, a high palatal vault, and a decrease in the distance between the lateral walls of the nasal cavity.^1^ The etiology is believed to be multifactorial^2^ and the main factors includes physiological problems, genetic and parafunctional habits^3^ that could lead to a mixed or mouth-breathing pattern.^4^ ^,^ ^5^ Since the airway is assumed to play a role in dentofacial development, the association with respiratory problems, especially nasal obstruction, has been the focus of many researchers who have investigated the possibility that these events are related.^6^ However, it could be considered erroneous to associate malocclusions only with the breathing pattern.^2^

The maxillary bones form approximately 50% of the nasal cavity’s anatomic structure.^7^ RME is a dentofacial orthopedic treatment procedure that has been widely used for correcting maxillary transverse deficiency in young patients and it can change the morphology of the maxillary arch, affecting the geometry and function of the nasal cavity.^7^ Eysel, in 1886, cited by Haas^8^ ^,^ ^9^ was the first rhinologist who studied the effect of RME on nasal cavity function. He found that, in the post-RME period, various changes occurred in the maxilla and adjacent bones, and RME caused an opening of the nasal cavity and reduction in nasal airway resistance. In addition, after the expansion an increase was found in the nasal cavity width and in the nasal base adjacent to the midpalatal suture. The maxillary sutures separate the external walls of the nasal cavity laterally, resulting in an increase in the intranasal capacity. Although orthodontic treatment is carried out to correct dental and skeletal discrepancies, some authors showed that RME outcomes could also be effective on naso-respiratory and sleep-disordered breathing problems of growing children.^11^ Therefore, RME has been suggested in the medical treatment of mouth-breathing, septal deformity, nasal infection, allergic rhinitis, and obstructive sleep apnea. However, its clinical use for rhinological effects is controversial.^12^ Its primary goal is to maximize orthopedic and minimize orthodontic tooth movement^13^ and there is an agreement among orthodontists that a maxillary constriction should be treated early,^9^ since the proportion of skeletal and dental movement depends on patient’s age and maturity level.^14^ ^,^ ^15^ In a study of RME effects, sex differences proved to be important as it is known that the facial skeleton significantly increases its resistance to expansion with increasing age and maturity.^15^

The majority of growth studies have used lateral cephalometric radiographs to analyze vertical and sagittal dimensional changes of the face. However, transverse problems and development of the oronasal area can be better understood by analyzing posteroanterior (PA) cephalometric radiographs.^5^ Structural remodeling of nasal cavity has the effect of increasing nasal patency after RME, and the relevance of PA radiograph is to show this remodeling and quantify the increase in nasal cavity,^16^ especially in the midface, since its walls are laterally displaced. The purpose of this article was to evaluate the skeletal effects of RME in the transverse and vertical dimensions on nasal cavity in mouth-breathing growing children and to correlate it with both sexes.

## MATERIAL AND METHODS

The study sample comprised 61 children (mean age 9.6 years, range 6.5 - 13.10 years; 35 male, 26 female) regardless of facial type or race, who sought treatment at the Department of Pediatric Otorhinolaryngology of the *Universidade Federal de São Paulo,* in Brazil. The criteria for selection of the treatment group were as follows: skeletal constricted maxillary arches, unilateral or bilateral posterior crossbite requiring RME treatment, and mouth-breathing. The exclusion criteria were: no maxillary first molars, metallic restorations, periodontal diseases, previous orthodontic treatment, and genetic disease involving chromosome or mutations.

In order to check for the mouth-breathing pattern, all patients were clinically examined by an experienced otorhinolaryngologist that verified the presence of nasal obstruction after anterior rhinoscopy, oroscopy and nasofiberendoscopy^17^. Potential candidates for adenoidectomy or adenotonsillectomy, with complete occlusion of the nasal cavity by nasal turbinates, intranasal tumors or polyps, adenoid occupying more than 70% of the choanas were excluded from the study.

The subjects were divided into two groups: males (mean age 10.1 years, range 6.5 - 13.10 years) and females (mean age 9.2 years, range 6.5 - 12.5 years). The study was approved by the Research Ethics Committee of *Universidade Federal de São Paulo* (protocol # 0907/08) and an informed consent was obtained from the parents or guardians, besides the verbal assent from the children before data collection for the study.

The orthodontic phase of treatment was undertaken at a local orthodontic clinics under the supervision of the department’s orthodontists. 

**Figure 1:**
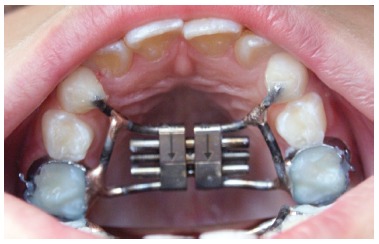
RME appliance, comprising a tooth-anchored device (conventional Hyrax expander).

Pretreatment (T_1_) orthodontic records, including PA radiographs were taken for all patients. Each patient underwent a standardized RME protocol using a tooth-anchored device activated by means of a modified Hyrax expander with a soldered framework and orthodontic bands on deciduous second molars and extended forward to the palatal surfaces of the deciduous canines (Fig 1); or supported by bilateral maxillary first premolars and first molars, in case the first premolars were sufficiently erupted. After the expander was cemented, it was activated six turns. Then, the parents or guardians were instructed to activate the jackscrew one turn (0.25 mm) twice a day until the required expansion was achieved. The degree of expansion was calculated for each patient, including a bilateral overexpansion and buccal tipping of a half-cusp width. After a mean active expansion period of 15 days (range 7- 21 days), the expander was tied off with a ligature wire and it was kept on the teeth as a passive retainer for at least 90 days (3 months), ranging from 91 to 106 days. This retention period allowed osseous reorganization of the midpalatal suture after expansion. All patients did not receive brackets or wires on the maxillary arch until T_2_ records were taken. 

**Figure 2:**
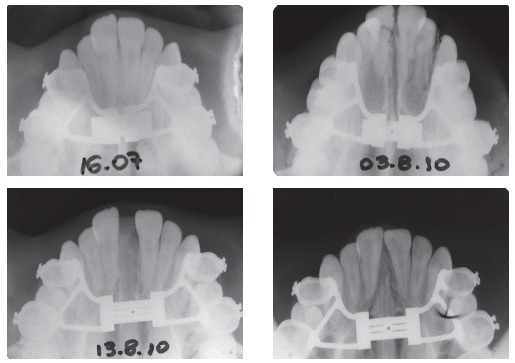
Pre-RME and post-RME occlusal radiographs.

**Figure 3:**
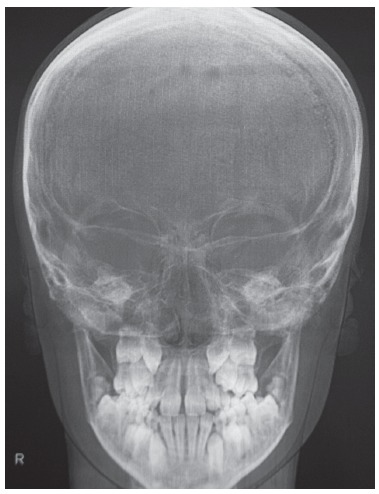
PA cephalometric radiograph pre-RME.

**Figure 4:**
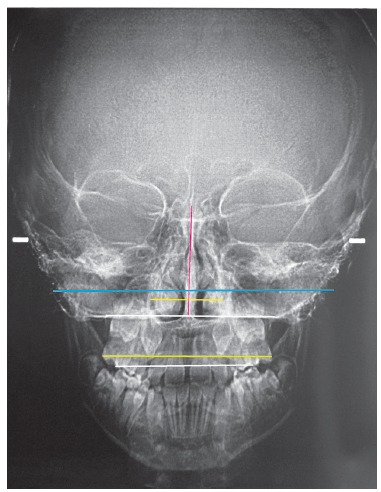
PA cephalometric radiograph post-RME, showing lines used for measurements.

Postexpansion (T_2_) PA cephalograms were taken after removal of the expander. The mean interval between T_1_ and T_2_ was 98 days (range, 91-105 days). As a result, study sample was composed by 122 PA cephalograms, which were hand traced with a 0.5-mm lead pencil on Ultraphan acetate tracing paper. All tracings were performed by the same investigator.

Tables 1 and 2 show the skeletal and dental landmarks, and linear measurements (in millimeters) from Ricketts’ cephalometric analysis.^17^

**Table 1:** Posteroanterior skeletal and dental landmarks.

Landmark	Description
NC/CN	The most lateral point of the nasal cavity
ZL/ZR	The most internal point of the frontozygomatic
ANS	Anterior nasal spine
JL/JR	Deepest point of the alveolar maxillary process
UML/UMR	The most prominent lateral point on the buccal surface of permanent first molar crown

**Table 2:** Posteroanterior planes and linear measurements.

Reference plane and line	Reference Plane Description
ZL-ZR	Z plane- reference line in the horizontal plane
Occlusal plane	Occlusal line in the molars
NC-CN	Nasal width
ANS-Z plane (NH)	Nasal height
JL-JR	Maxillary width
IM	Intermolar width - distance between UML-UMR parallel to the occlusal plane.
IM/ JL-JR	Ratio between intermolar width and maxillary width
JL-JR/ NC-CN	Ratio between maxillary width and nasal width

### Statistics analysis

Descriptive statistics including mean, standard deviation (SD), and ranges were calculated for T_1_ and T_2_ measurements. Independent samples *t*-test was performed for each measurement, to determine sex differences. Significance for all statistical tests was predetermined at *p*< 0.05. T_1_and T_2_ measurements and comparisons are presented in Tables 3 to 6. To estimate intraexaminer error, reliability and reproducibility, 20 randomly selected records (10 males and 10 females) were reevaluated after approximately 6 months from preliminary data collection. All parameters were measured by the same observer (intraexaminer error). Normality was assessed with the Shapiro-Wilk test (*p*> 0.05). After that, Student’s *t*-tests were used to investigate the difference between both measurements, and intraclass correlation coefficient (ICC) was used to test reliability. Statistical treatment of the data was performed using Statistical Package for the Social Sciences (SPSS), version 22 for Windows. All statistical analyses were performed using SPSS v. 16.0 (SPSS Inc, IL, USA) software.

**Table 3:** Comparisons of T1 values between males and females.

Variable	Sex	Mean	n	SD	P
Age (month)	Males	121.5	35	24.8	0.070
	Females	108.2	26	31.5	
Height (mm)	Males	53.9	35	3.4	0.025*
	Females	51.6	26	4.3	
Nasal width (mm)	Males	28.7	35	2.8	0.782
	Females	28.5	26	3.2	
Área (nasal width x nasal height) (mm^2^)	Males	1551.8	35	206.5	0.153
	Females	1473.1	26	214.5	
Maxillary width (mm)	Males	63.6	35	3.8	0.026*
	Females	61.4	26	3.6	
Intermolar width (mm)	Males	58.3	35	3.6	0.239
	Females	57.2	26	3.7	
Intermolar width/ Maxillary width ratio	Males	1.09	35	0.06	0.426
	Females	1.08	26	0.08	
Maxillary width /Nasal width	Males	2.23	35	0.19	0.267
	Females	2.17	26	0.20	

NS = nonsignificant; n = number of patients; SD = standard deviation. *Statistically significant (*p* < 0.05).

**Table 4:** Comparisons of T_2_ measurements between males and females.

Variable	Sex	Mean	n	SD	P
Height (mm)	Males	56.4	35	3.65	0.05*
	Females	54.2	26	4.79	
Nasal width (mm)	Males	31.3	35	3.27	0.543
	Females	31.9	26	3.61	
Área (nasal width x nasal height) (mm^2^)	Males	1770.7	35	248.6	0.548
	Females	1730.8	26	263.1	
Maxillary width (mm)	Males	67.4	35	3.32	0.007**
	Females	64.7	26	4.05	
Intermolar width (mm)	Males	65.4	35	3.44	0.029*
	Females	62.9	26	5.08	
Intermolar width/ Maxillary width ratio	Males	1.03	35	0.04	0.996
	Females	1.03	26	0.07	
Maxillary width / Nasal width	Males	2.17	35	0.18	0.014*
	Females	2.05	26	0.18	

NS = nonsignificant; n = number of patients; SD = standard deviation. Statistically significant, *p < 0.05, **p < 0.01.

**Table 5:** Comparisons of changes from T_1_ to T_2_, between males and females.

Variable	Sex	Mean	n	SD	P
Differences in height (mm)	Males	2.4	35	1.45	0.750
	Females	2.6	26	1.82	
Differences in nasal width (mm)	Males	2.6	35	1.40	0.027*
	Females	3.4	26	1.20	
Differences in area (nasal width x nasal height) (mm^2^)	Males	218.8	35	93.9	0.103
	Females	257.7	26	86.0	
Differences in maxillary width (mm)	Males	3.8	35	3.11	0.550
	Females	3.3	26	2.12	
Differences in intermolar width (mm)	Males	7.1	35	3.15	0.124
	Females	5.8	26	3.41	
Differences in intermolar width/Maxillary width ratio	Males	-0.06	35	0.06	0.237
	Females	-0.04	26	0.05	
Differences in maxillary width/Nasal width	Males	-0.06	35	0.10	0.019*
	Females	-0.12	26	0.10	

n = number of patients; SD = standard deviation. **Statistically significant (*p* < 0.01).

**Table 6:** Changes from T_1_ to T_2_ within the groups of males and females.

Variable	Mean	N	SD	P
Differences in height (mm)	2.5	61	1.6	0.000**
Differences in nasal width (mm)	2.9	61	1.4	0.000**
Differences in area (nasal width x nasal height) (mm^2^)	235.4	61	91.9	0.000**
Differences in maxillary width (mm)	3.6	61	2.7	0.000**
Differences in intermolar width (mm)	6.5	61	3.3	0.000**
Differences in intermolar width/Maxillary width ratio	-0.05	61	0.06	0.000**
Differences in maxillary width/Nasal width	-0.09	61	0.10	0.000**

NS = nonsignificant; n = number of patients; SD = standard deviation. *Statistically significant (*p* < 0.05).

### Normality of data

The normality of data was assessed by the Shapiro-Wilk test. Considering a significance level of 5%, there were no significant deviations from the normality of the data (*p*> 0.05), neither in T_1_ nor in T_2_. For this reason, the following parametric tests were used to analyze the error and reliability of the measurements: Student’s *t*-test for paired samples and Intraclass Correlation Coefficient (ICC).

### Sample calculation

In this study, the effect size of Student’s *t*-tests was calculated to verify the suitability of the sample for independent samples (comparisons for the sexes) and paired samples (comparison between T_1_ and T_2_), with a power of 80 % and a significance level of 5%. The classifications for effect size proposed by Cohen^19^ were considered: D = 0.2, small effect; D = 0.5, mean effect; D = 0.8, high effect. The calculations were performed with the G * Power software.^20^

Under these conditions, the sample allowed to detect mean effects (Cohen’s D = 0.65) in the comparison between the male group (n = 35) and the female group (n = 26). For the comparison between T_1_ and T_2_ (n = 61), the sample allowed to detect small effects (Cohen D = 0.32).

### Method error analysis

The results obtained through the Student’s *t*-test for paired samples showed correspondence between the initial measurements and the repetitions by the same evaluator (Intraclass Correlation Coefficient), both in T_1_ (*p*> 0.05 and ICC close to 1.000) and in T_2_ (*p*> 0.05 and ICC close to 1.000), indicating the absence of measurement error, reliability and reproducibility (Table 7).

**Table 7:** Error analysis: mean and standard deviation (SD), Student’s *t*-test for paired samples and ICC (n = 20).

Variable	Sex	Measurement T_1_ Mean (SD)	Measurement T_2_ Mean (SD)	Student’s t-test	ICC
Nasal width	Males	21.78 (1.64)	23.74 (2.68)	0.531	0.989
	Females	22.22 (1.71)	22.12 (1.56)	0.671	0.907
Height	Males	42.77 (3,99)	45.94 (4.29)	0.477	0.999
	Females	41.0 (4.09)	40.83 (4.01)	0.267	0.993
Intermolar width	Males	102.55 (5.41)	104.16 (5.19)	0.443	0.999
	Females	101.21 (4.19)	101.3 (4.42)	0.653	0.991
Maxillary width	Males	58.72 (2,86)	61.93 (3.42)	0.067	0.998
	Females	58.79 (3.03)	58.71 (3.01)	0.070	0.998

SD = standard deviation. ** Statistically significant (*p* < 0.05).

## RESULTS

Table 3 refers to sex differences in T_1_ and it shows statistical significance for nasal height and maxillary width variables, which were smaller in female group.

Table 4 compares the sex differences at T_2_. RME produced greater increases in the male group for nasal height, maxillary width, intermolar width and nasal width/maxillary width ratio. 

Table 5 compares the sex differences at T_1_ and T_2_. Significant changes in the nasal width were observed in female group, and the nasal width/maxillary width ratio was smaller in female group.

Table 6 shows changes within the groups (T_2_-T_1_). RME produced skeletal, dental and nasal increases in both males and females.

All measurements were considered to be reliable, since the reliability statistics were equal to 1, which indicates perfect reliability.

## DISCUSSION

In both males and females groups, the maxilla showed a significant increase for the linear measurements after RME, which is in accordance to the findings of other authors that reported post-RME sagittal and transverse increases of skeletal, dental and nasal structures.^3^ ^,^ ^15^ ^,^ ^21^ ^-^ ^23^ According to Wriedt et al^24^ and Warren et al,^25^ the enlargement of the nasal valve with an increase of nasal volume can result in improvement of nasal breathing. More controversial is the question of whether RME can achieve a shift from mouth to nasal breathing patterns and change the subjective sensation of nasal obstruction.^26^ These effects depend on the existence or not of nasal obstruction and on its cause, location and severity.^6^

Statistical results in Table 3, which compares the values obtained in the pre-RME (T_1_) between males and females, show that most of the variables were not significant (*p*> 0.05) indicating no correlation between sex and the linear measurements in T_1_. The two statistically significant variables - nasal height (*p*= 0.025) and maxillary width (*p*= 0.026) -for males are in agreement with other studies in the literature^5^. Snodell et al^5^ showed sex differences in maxillary width at 6 years old and higher values for maxillary width and intermolar width at age 12 years in males. In the composition of the mean values of the nasal cavity found by Ricketts et al,^18^ the proportion between nasal height and nasal width represents 60%. In this study, this proportion was 56% in both males and females groups, confirming reduced nasal width in the mouth-breathing sample. Whereas the growth in the vertical direction is larger than in the transverse one, from 6 to 18 years of age - with a increased percent in males - and by evaluating the mean age of the sample, the proportion between the values of transverse and vertical measures was below the one found in longitudinal studies of facial growth.^5^ These results are consistent with studies reporting a decrease in measures of the nasal cavity in mouth-breathing patients.^6^

The results in Table 4 refer to the sex difference at T_2_. RME produced greater increases in the male group for nasal height (*p*= 0.05), maxillary width (*p*= 0.026), intermolar width (*p*= 0.029) and nasal width/maxillary width ratio (*p*= 0.014). The maxillary width increase correlates with intermolar width, according to longitudinal cephalometric studies, and the nasal width was also correlated with maxillary width and maxillary intermolar width.^5^ The greatest increase in the maxillary width and in the intermolar width in males can be understood by the higher bone resistance of females, which complete puberty earlier than males, and the higher maxillary growth rate in males, which may affect resistance to the expansion forces,^27^ leading to increased expression of dental effects in males. For this reason, timing orthodontic treatment to coincide with growth may be a considerable factor in the stability of the dentition, and studies agree that the use of orthopedic maxillary expansion should be cautiously applied past the age of 15 years for females and 17 years for males.^28^

The nasal width was correlated with maxillary width for both males and females, indicating the relationship between airway and maxillary width.^5^ More than 80% of the maxillary growth is completed at age 6 to 11 years, with a higher rate in females; excluding nasal width - with a higher rate in males, which is only 75% complete in females,^5^ justifying the greater increase in nasal width in females found in the present study. The significant maxillary width/nasal width ratio value shows transverse growth of the nasal cavity and control of maxillary constriction, which are important for dental and skeletal relationships and nasal airway increase. The arch width and arch depth are dependent on each other;^29^ thereby, the increase of the nasal floor close to the midpalatal suture in width after RME and the growth in the vertical direction with a higher rate in males^5^ - according to longitudinal studies of facial growth - could explain their nasal height increase.

The analysis between males and females at T_1_ and T_2_ is shown in Table 5, which compares the mean values of all variables. The only two significant variables were: nasal width (*p*= 0.027) and nasal width/maxillary width ratio (*p*= 0.019). Negative values in the nasal width/maxillary width ratio and the radiographic evaluation in frontal and occlusal norms confirm that the maxilla was laterally moved, creating a pyramidal shape with the fulcrum located near frontonasal suture and PNS, with a larger opening in the anterior maxillary base.^8^

Studies have shown the benefits of RME for the enlargement of the nasal cavity and improvement of nasal permeability through otorhinolaryngologist evaluation, radiographic examination, rhinomanometry, acoustic rhinometry,^7^ ^,^ ^11^ ^,^ ^12^ ^,^ ^30^ ^-^ ^35^ and subjective evaluation conducted among patients reporting improved breathing.^6^ ^,^ ^17^

Many studies have emphasized the ability of RME to produce lateral expansion of the nasal cavity and to decrease nasal resistance^6^ ^,^ ^7^. According to the present study results, the use of RME showed favorable results in the treatment of maxillary constriction and increased nasal cavity for both sexes; thereby, a possibility is offered that some potential may exist for a young patient to “outgrow” a breathing problem.

The Table 6 shows the effects of RME on the skeletal, dental and nasal structures at T_1_ and T_2_. The results show that both males and females were significantly favored (*p*< 0.01) by the transverse increase of the maxilla and nasal cavity. After statistical analysis and comparison of measurements in males and females, all linear variables were not significant. The transversal changes were not significantly different between sexes in the treatment of maxillary constriction with RME for a sample with a mean age of 9 years and 7 months.

As this was a short-term study, the alterations observed in the pretreatment and immediate posttreatment radiographs represent the effects produced by the expander. It is important to point out that the time interval between the first and the second radiograph in this study did not exceed three months, which minimizes growth as a variable in the interpretation of the results. Therefore, studies of facial growth had to be considered in the interpretation of the differences between sexes.

## CONCLUSION

There was no evidence that maxillary constriction in growing subjects is in any way associated with sex.

The treatment of maxillary constriction with RME in mouth-breathing patients without nasal obstruction showed significant improvement in the nasal width/maxillary width ratio, representing a transverse increase of the nasal cavity.
